# Enhancing Quality of Life: Pre- and Postoperative Assessment in Idiopathic Hyperhidrosis Patients

**DOI:** 10.7759/cureus.49588

**Published:** 2023-11-28

**Authors:** Cesar Estrella-Gaibor, Yeisson Rivero, Flor Jaramillo-Montaño, Livan Veitia, Jesus Cordova Guilarte, Andrea Garcia

**Affiliations:** 1 General Surgery, Ministerio de Salud Pública, Hospital Esmeraldas sur Delfina Torres de Concha, Quito, ECU; 2 Surgery, Universidad de Oriente, Barcelona, VEN; 3 Pediatric Surgery, Hospital Instituto Ecuatoriano de Seguridad Social (IESS) Esmeraldas, Quito, ECU; 4 Surgery, Hospital Pediátrico Universitario de Centro Habana, La Habana, CUB; 5 Medicine, Universidad de Oriente, Barcelona, VEN; 6 Internal Medicine, Universidad de Oriente, Barcelona, VEN

**Keywords:** compensatory hyperhidrosis, postoperative complications, quality of life, sympathectomy, hyperhidrosis

## Abstract

Objective: This study aimed to characterize patients, describe surgical complications, and evaluate the pre- and postoperative quality of life (QOL) of individuals who underwent surgery for primary hyperhidrosis (PHH).

Methods: A prospective, non-randomized, uncontrolled study was conducted, documenting cases of patients undergoing surgery for PHH at a reference center in La Habana, Cuba, from January 2016 to December 2022.

Results: A total of 49 cases were described, with a median age of 16 years; 59.1% were female. The most common presentation was palmar-plantar-axillary, observed in 53% of cases. The palmar presentation was more frequent in female patients (p<0.05). Within 24 hours post-procedure, 85.7% of patients showed dryness in the palmar and axillary areas, with surgical complications occurring in 14.3% of cases (intercostal neuritis, pneumothorax, and hemothorax). CH of some form was recorded in 89.8% of cases. At least 30 days after the surgery, 95.9% of the patients reported a significant improvement in their QOL.

Conclusion: Thoracic sympathectomy is an efficient and safe method for treating hyperhidrosis in adolescents, leading to an enhanced QOL. However, this study reported a higher incidence of complications, particularly CH, compared to previous national and international studies.

## Introduction

Primary hyperhidrosis (PHH) is a non-organic benign disease characterized by excessive sweating on the local palm, which may also be accompanied by hyperhidrosis in other locations, including the craniofacial region, axilla, chest, abdomen, back, and planta [1]. It involves sweating more than expected for environmental conditions and thermoregulation over a span of at least six months. The diagnosis of PHH is by definition non-association with any condition, and other causes need to be excluded. Secondary hyperhidrosis, in contrast, is attributed to numerous other diseases including endocrine disorders, drugs, malignant diseases, and abnormalities of the central nervous system. In both cases, the diagnosis is eminently clinical [2].

The plantar forms could generate local infections, bromhidrosis, irritant dermatitis, and blisters that deteriorate socks and footwear, in addition to producing an unpleasant odor. Axillary hyperhidrosis causes wetting, staining, and deterioration of clothing, as well as bad odor. However, the palmar shape is the most problematic from a social point of view and the one that is most likely to require specialized consultation [3]. There is a wide variation in the prevalence of PHH worldwide, from as low as 0.93% to as high as 14.7% [4].

Hyperhidrosis generates patient discomfort, embarrassment with social exposure, and impairment in social and emotional relationships, which significantly affects the quality of life (QOL) of patients [5]. In addition, it alters domains such as nutrition, perception/cognition, self-perception, role/relationships, coping/stress tolerance, safety/security, and comfort [6]. These alterations directly affect their QOL, which is defined by the World Health Organization as the perception an individual has of his or her place in existence, in the context of the culture and value system in which he or she lives, and in relation to his or her goals, expectations, norms, and concerns [7].

In children, PHH affects school activities and causes stationery to get wet; in team sports, in games, and in daily life, they suffer the rejection of their peers and family before contact with wet hands. The great embarrassment comes from having soaked clothes and having to change them two or more times a day. They avoid handshakes when possible. Continuous moisture and exposure to bacteria lead to the maceration of the skin, accompanied by fungal infections [8].

There are non-surgical treatments, but all of them only have palliative potential, and when interrupted, the symptoms reappear in almost all patients, including anticholinergic drugs (oxybutynin, propantheline), which usually carry side effects [2].

Endoscopic thoracic sympathectomy (ETS) is by far the best treatment choice for PHH with the most stable and durable curative effects (level of evidence: 1; strength of recommendation: strong). ETS is a method to decrease the sympathetic nervous impulse to the sweat glands by cutting off the thoracic sympathetic chain through which their secretion is controlled. The purpose of the method is to totally or partially eliminate/disconnect the thoracic sympathetic nodes [9].

In Cuba, the first thoracoscopy sympathectomies were performed at the National Center for Endoscopic Surgery in La Habana in 2006, where five cases were treated with a 90% cure rate [10]. In 2009, more than 50 patients were operated on at the same center. Interventions for pediatric patients began in 2011 [11]. PHH is a novel, studied, and undervalued entity in the Cuban population. Therefore, the present study aimed to assess the pre- and postoperative QOL of patients with PHH after the ETC, as this last aspect of the patient’s health has not been included in detail in previous studies with Cuban patients. Furthermore, we aimed to characterize the patients, identify the presence of compensatory sweat, describe the surgical complications, and identify the most affected spheres (social, emotional, and functional) in the context of their surgical resolution.

## Materials and methods

This was an observational study with the general objective of evaluating the pre- and postoperative QOL of all patients who underwent surgery for PHH at a reference center in La Habana, Cuba, from January 2016 to December 2022. The study population consisted of 49 patients, including those over 12 years of age, who underwent PHH surgery using the video thoracoscopic sympatholytic technique through a single port with apneic oxygenation. The voluntary consent of parents and/or legal guardians for the participation of patients in the research was obtained through the signing of the informed consent document. The Medical Ethics Committee of Centro Habana Pediatric Hospital approved the study.

All patients underwent ETS using the same surgical technique: patients were positioned semi-seated at a 30-degree angle with their arms in abduction for the administration of general anesthesia via conventional orotracheal intubation. Basic cardiorespiratory monitoring was applied, and temperature was measured in both hands. A single 10 mm incision was made along the axillo-mammary line, providing access to the hemithorax up to the level of the third intercostal space at the clavicular midline. A 10 mm lens with a working channel (one hole) was utilized, and lung collapse was achieved through apneic oxygenation, ensuring adequate exposure of the surgical field. Insufflation of CO^2^ was unnecessary. A preganglionic sympathetic nerve section (sympathicotomy) was performed at the T3 and T4 levels using monopolar electrofulguration, extending 2 cm along the midline of the rib to eliminate potential nerve branches such as Kuntz branches that may be present in some cases. Total lung expansion was visualized, and the trocar was removed without the need for a chest tube. Bilateral thoracic approaches were carried out sequentially within the same anesthetic timeframe, always commencing on the right side. Postoperative antibiotic therapy was not administered, and chest X-ray controls were not conducted. Children were discharged the following day if they did not experience any complications.

Data were collected from medical records using a data collection form. Additionally, the QOL survey, as used by Ribas-Milanés to assess QOL before and after thoracic sympathectomy, was administered either in person or over the telephone one week before the surgery and from 24 hours to 30 days after the procedure. Patients were accompanied by their parents at all times. Patients with incomplete assessments were not included. The questionnaire comprised 20 questions divided into five specific domains. For the purposes of this study, the overall assessment of QOL before and after surgery featured five response levels based on tables that permitted only one answer. Specific domains (social, personal, etc.) were answered dichotomously, indicating the presence or absence of any alterations in each category [12,13]. Quantitative variables were expressed as medians. The McNemar test was used to evaluate differences before and after the surgery. An exploratory analysis between variables was performed using the chi-square test. All data were tabulated in a Microsoft Excel database (Microsoft, Washington, USA). The results are presented in tables displaying absolute and relative frequency distributions.

## Results

Data from a total of 49 cases was collected and analyzed between the years 2016 and 2022. The age of the patients ranged from 13 to 18 years. The median age of the studied population was 16 years, with 59.1% being female. All patients were in good health, with no notable medical history or physical examination findings.

The most common presentation was palmar-plantar-axillary in 47% of cases (n=23), followed by palmar-axillary in 28.6% (n=14), only palmar in 14.3% (n=7), and palmar-plantar in 10.2% (n=5).

Following the ETS procedure within the first 24 hours, the following observations were made: an increase in hand temperature in 100% of cases, palmar and axillary dryness in 85.7% (n=42), and improvement in plantar sweating in 30.6% (n=15). Only one case showed no changes following the operation.

Surgical complications occurred in 14.3% (n=7) of cases, listed in order of frequency: intercostal neuritis in 6.1% (n=3), pneumothorax in 4.1% (n=2), hemothorax in 2% (n=1), and pneumonia in 2% (n=1). Compensatory hyperhidrosis (CH) of some kind was recorded in 89.8% of cases (n=44). The most common location of PHH was the back in 38 patients, followed by the abdomen (n=37), thighs (n=21), legs (n=2), and upper lid (n=2).

A significant aspect of this study focused on evaluating the QOL of the patients. The results of the preoperative answers to the questionnaire related to their overall evaluation of QOL are shown in Table 1. It can be noted that all patients were in a bad or very bad condition. The results of the patients' QOL at least 30 days after surgery are shown in Table 2. It can be noted that all of the patients experienced a positive change in their QOL after the procedure.

**Table 1:** Overall evaluation of QOL related to hyperhidrosis before surgery QOL: quality of life

QOL	Number (49)	Percent
Good	1	2
Poor	29	59.2
Very poor	19	38.8

**Table 2:** Overall evaluation of QOL related to hyperhidrosis after surgery QOL: quality of life

QOL	Number (49)	Percent
Much better	47	95.9
Slightly better	2	4.1

The presence of alterations in various aspects of QOL before and after surgery is detailed in Table 3. Notably, for the categories "wearing sandals/walking barefoot" and "wearing warm clothes," the improvements were not absolute but remained statistically significant.

**Table 3:** Pre- and postoperative assessment in the specific domains of QOL ^a ^The difference was statistically significant (p<0.05) using the McNemar test

Domains	Before surgery	After surgery	p-value
Social/functional	n (%)	n (%)	
Writing and manual work	49 (100)	1 (2)	-
Leisure	35 (71.4)	0 (0)	-
Sport	30 (61.2)	0 (0)	-
Handshaking	49 (100)	1 (2)	-
Socializing	20 (40.8)	0 (0)	-
Grasping objects	49 (100)	1 (2)	-
Social dancing	15 (30.6)	0 (0)	-
Personal			
Holding hands	49 (100)	0 (0)	-
Intimate touching	32 (65.3)	0 (0)	-
Intimate affairs	14 (28.6)	0 (0)	-
Emotional sphere			
Self-punishment	25 (51)	0 (0)	-
Rejection of people	19 (38.8)	0 (0)	-
Other situations			
When wearing warm clothing	20 (40.8)	9 (18.4)	0.000977 ^a^
When tensed or worried	49 (100)	0 (0)	-
When thinking about illness	41 (83.7)	0 (0)	-
When being in public	12 (24.5)	0 (0)	-
When wearing sandals/walking barefoot	24 (49)	13 (26.5)	0.003418 ^a^
Had problems because of the illness at school or work	38 (77.6)	0 (0)	-

An exploratory analysis was conducted among the different variables. It was observed that palmar presentation was more frequent in female patients compared to male patients (p<0.05). There was no relationship found between the patient's sex and the incidence of postoperative complications, as shown in Table 4.

**Table 4:** Differences in clinical presentation and complications according to sex ^a^ Fisher's exact test ^b^ Chi-square test

Presentation	Female	Male	p-value
Palmar	7	0	0.03634 ^a^
Palmar-plantar	3	2	>0.9999 ^a^
Palmar-axillary	5	9	0.03451 ^b^
Palmar-plantar-axillary	14	9	0.82130 ^b^
Complications	5	2	0.47648 ^b^
Compensatory hyperhidrosis location			
Thigh	15	6	0.13096 ^b^
Back	20	18	0.08284 ^b^
Abdomen	20	17	0.19954 ^b^
Legs	1	1	0.78730 ^b^
Upper lip	2	0	0.23046^ b^

## Discussion

An analysis was conducted on a cohort of adolescent patients with hyperhidrosis before and after surgical management, resulting in a high rate of hyperhidrosis resolution and a significant improvement in the QOL. However, a notable incidence of complications was observed.

Hyperhidrosis is most commonly observed during adolescence and early young adulthood, typically occurring between the ages of 14 to 15 years and up to 25 years. This is attributed to the increased development of sweat glands and heightened sweat production during this phase of life. In our study, we noted that patients between the ages of 16 and 18 were the most prevalent, aligning with findings reported by Park et al. in 2010, where they also observed an elevated incidence of hyperhidrosis in patients over the age of 14 [14].

Regarding gender distribution, our case material demonstrated a predominance of female patients. This observation aligns with findings from a study by Hasimoto et al., which reported hyperhidrosis prevalence in Brazil and encompassed patients aged 5 to 97 years, revealing that 60% of those patients were female [4]. A similar study conducted in the United States also noted a higher proportion of female patients, with 53% of individuals diagnosed with hyperhidrosis being female [15]. This gender disparity persists even within the pediatric population [16].

Despite the overall predominance of hyperhidrosis in females, some studies have indicated that, when it comes to palmar hyperhidrosis specifically, males may be more significantly affected than females [17]. In our study, however, we found a statistically significant difference, indicating that palmar hyperhidrosis was more common among females. This difference could be associated with the specific age groups that were studied.

It's possible that the higher incidence of hyperhidrosis in women is partly attributed to underreporting among men. Women may be more inclined to seek treatment due to aesthetic concerns, which could lead to a more accurate diagnosis in females. Moreover, the higher prevalence of anxiety in women compared to men may also contribute to the increased occurrence of hyperhidrosis in females, as anxiety is a recognized trigger for excessive sweating [18].

In this study, the most common form of hyperhidrosis presentation was the combination of palmar, plantar, and axillary hyperhidrosis. Similar results were reported by Osorio et al., who found that the same areas were the most frequent locations of hyperhidrosis in a cohort of adolescents who were followed up for surgical resolution 10 years later [19]. On a broader scale, it's worth noting that 20% of individuals suffering from hyperhidrosis report involvement in a single isolated area, while 65% experience axillary hyperhidrosis (with 10% being isolated and 55% combined with other locations, with the most common combination being facial hyperhidrosis in 29%) [15]. The axillary and palmar regions are consistently reported as the most frequently affected sites of sweating in the literature [14,19].

The location of sweating is important, as it has been shown to be a risk factor for CH. Carvalho et al. found that axillary hyperhidrosis appears to be associated with the development of dorsolumbar CH [20]. This could partially explain the high incidence of CH in our study, where axillary hyperhidrosis was among the most common locations.

Thoracoscopic sympathectomy is a minimally invasive and safe procedure that delivers excellent therapeutic outcomes with a low recurrence rate. Patients benefit from rapid pain relief, short hospital stays, and favorable cosmetic results. This technique has consistently yielded significant results and is consistently recommended as the treatment of choice by authors such as Buraschi et al. [21] and Kuijpers et al. [22], who reported a success rate of 98% and 97%, respectively. Similar to our study, where we recorded a 98% success rate, this rate aligns with previous reports in Cuba [23].

The level of sympathectomy is a critical factor in determining postoperative outcomes, including the complete elimination of hyperhidrosis and the occurrence of CH, which can significantly impact postoperative QOL [24]. At our center, we perform ETS at the T3 and T4 levels to address palmar and axillary sweating. This technique has proven successful in achieving palmar and axillary anhidrosis in the majority of patients, a result also reported in studies conducted by Zacarías et al. [23] and Soto et al. [11] in Cuban centers, as well as in other series involving children [25].

The primary objective of this procedure is to enhance the QOL while keeping complications to a minimum and essentially eliminating them. The most prevalent side effect of hyperhidrosis surgery is CH, with reported occurrences in the literature ranging from 3% to 98% [24]. In our study, the majority of patients (89.8%) experienced this complication. Previous studies conducted in Cuba reported an incidence rate of 24.3% in a group of 37 children who underwent the procedure between 2011 and 2012 [11] and 48.1% in a series of 276 patients aged 14 to 60 who were operated on between 2011 and 2015 [23]. This reflects an increasing trend in the rate of this complication, at least at the local level, despite the surgical interventions being performed at the same level of spinal roots, a factor that must be considered the most commonly cited risk factor in the literature for moderate to severe CH is the interruption of the T2 ganglion (T2-T3 level) [24].

Regarding the location of the CH, the study by Soto et al. aligns with our findings, with the back and abdomen being the most common areas affected [11]. In Latin America, studies like that of Zamarin et al. reported rates as high as 78% in patients, affecting primarily more than one body segment, with the back being the most affected [5].

On an international scale, in a study involving 58 patients under 25 years of age in Belgium, the rate of post-ETS CH was 15.5% [25]. In the study by Kuijpers et al., with 25 patients, less than 27% developed CH, and no severe cases were reported [22]. On the other hand, in the United States, Gunn et al. reported that 29% experienced transient CH, and 4% complained of severe CH, requiring further treatment [26].

This wide variability in the incidence of CH may be attributed to heterogeneous patient populations or variations in surgical procedures.

Other less common complications include pneumothorax requiring chest tube drainage (1%), pleural effusion (1%), acute bleeding or delayed hemothorax (1%), chylothorax, and persistent intercostal neuralgia (<1%) [24]. In this cohort of patients, 14.3% experienced complications, with intercostal neuritis being the most common, present in three patients. Similar to CH, there is an observed increase in the percentage of these types of complications. In Cuba, the study by Soto et al. did not report any of these complications [11], while the study by Zacarías et al. recorded complications in 1.1% of patients [23]. Even in the study by Zamarin et al. in Chile, the rate of these complications was 3.2% [5]. Among these studies, bleeding was the most common complication, whereas, in our registry, there was only one case of hemothorax. In Italy, Ibrahim et al. reported rates between 6% and 8% of unilateral hemothorax seven days after single-port video-assisted thoracoscopic sympathectomy [27].

In adolescents, hyperhidrosis can have a significant impact on various aspects of their social, emotional, and personal lives. In Cuba, previous studies on hyperhidrosis did not directly address the QOL of patients before and after the intervention. In our study, patients reported a poor or very poor preoperative QOL, but all of them experienced an improvement in their QOL after the procedure. This improvement is a common result observed in different studies, such as the one by Milanez et al., where 86.4% of patients reported a better QOL after the procedure in Brazil [12]. However, contrasting results were found in other studies, like the one by Zamarin et al., where although most patients showed postoperative improvement, the contrast was not as pronounced as in our study, due to lower preoperative impairment among their patients and more postoperative cases with "same" or "worse" assessments [5]. A similar trend was observed by Hasimoto et al., where 48% of patients considered themselves to have good QOL before the intervention [4].

Among the evaluated domains (Table 1), the most affected were the special conditions and functional-social domains. All patients experienced difficulties in writing, performing manual tasks, grasping objects, and shaking hands. Furthermore, all patients also expressed dissatisfaction due to sweating when they felt tense or anxious. Similar impairments in these domains before surgery were found in the sample studied by Hasimoto et al. [4].

Although our study had a high rate of CH, it did not significantly impact the postoperative QOL of the patients, with most of them reporting a much better QOL after the procedure, similar to previous studies [5,12].

Regarding its limitations, this study did not include the Hyperhidrosis Disease Severity Scale (HDSS) [28] or assess the satisfaction level after the intervention, which is common in various studies on the topic. Nevertheless, the subjective evaluations of QOL before and after the surgery presented in this study provided an indirect assessment of both aspects.

The study was conducted at a specific center in La Habana, Cuba, with a non-randomized and uncontrolled design. The relatively higher rate of complications observed compared to previous research suggests the need for caution when applying these findings to different populations and healthcare settings. Further research in diverse contexts is necessary to confirm these results' generalizability.

## Conclusions

Thoracic sympathectomy is a straightforward, efficient, and safe method for treating hyperhidrosis in adolescents, leading to an enhanced QOL for patients who can experience significant challenges before the procedure. While the majority of patients with this condition are female, there is a wide variation in these statistics across studies. A higher incidence of complications, particularly CH, was reported in the group of patients under investigation compared to previous national and international studies.
